# Chronic Glutamate Toxicity in Neurodegenerative Diseases—What is the Evidence?

**DOI:** 10.3389/fnins.2015.00469

**Published:** 2015-12-16

**Authors:** Jan Lewerenz, Pamela Maher

**Affiliations:** ^1^Department of Neurology, Ulm UniversityUlm, Germany; ^2^Cellular Neurobiology Laboratory, Salk Institute for Biological StudiesLa Jolla, CA, USA

**Keywords:** glutamate receptors, glutamate transporters, system x^−^_*c*_, Alzheimer's disease, Huntington's disease, amyotrophic lateral sclerosis, neurodegeneration, excitotoxicity

## Abstract

Together with aspartate, glutamate is the major excitatory neurotransmitter in the brain. Glutamate binds and activates both ligand-gated ion channels (ionotropic glutamate receptors) and a class of G-protein coupled receptors (metabotropic glutamate receptors). Although the intracellular glutamate concentration in the brain is in the millimolar range, the extracellular glutamate concentration is kept in the low micromolar range by the action of excitatory amino acid transporters that import glutamate and aspartate into astrocytes and neurons. Excess extracellular glutamate may lead to excitotoxicity *in vitro* and *in vivo* in acute insults like ischemic stroke via the overactivation of ionotropic glutamate receptors. In addition, chronic excitotoxicity has been hypothesized to play a role in numerous neurodegenerative diseases including amyotrophic lateral sclerosis, Alzheimer's disease and Huntington's disease. Based on this hypothesis, a good deal of effort has been devoted to develop and test drugs that either inhibit glutamate receptors or decrease extracellular glutamate. In this review, we provide an overview of the different pathways that are thought to lead to an over-activation of the glutamatergic system and glutamate toxicity in neurodegeneration. In addition, we summarize the available experimental evidence for glutamate toxicity in animal models of neurodegenerative diseases.

## Introduction

L-glutamate (L-glu) is the major excitatory neurotransmitter in the brain and is functionally involved in virtually all activities of the nervous system. In this section, we will first outline the general principles of L-glu signaling in the brain. Then, we will broaden this scheme by expanding the model to (a) different pools of extracellular glutamate (synaptic, perisynaptic, and extrasynaptic) resulting from either vesicular and non-vesicular sources or atypically located glutamate receptors outside of synapses and (b) other molecules present in the brain that have the ability to activate glutamate receptors and discuss their possible physiological functions.

### Glutamate signaling in the brain

The human brain contains 6–7 μmol/g wet weight of L-glu (Perry et al., 1971; Lefauconnier et al., 1976). Thus, L-glu along with glutamine is the most abundant free amino acid in the central nervous system. More than five decades ago, Curtis et al. demonstrated that L-glu has an excitatory action on nerve cells (Curtis et al., 1960). Since then, its role as an excitatory neurotransmitter as well as its cerebral metabolism have been studied in detail (reviewed by Fonnum, 1984; Hertz, 2006; Marmiroli and Cavaletti, 2012; Zhou and Danbolt, 2014).

**Figure 1:**
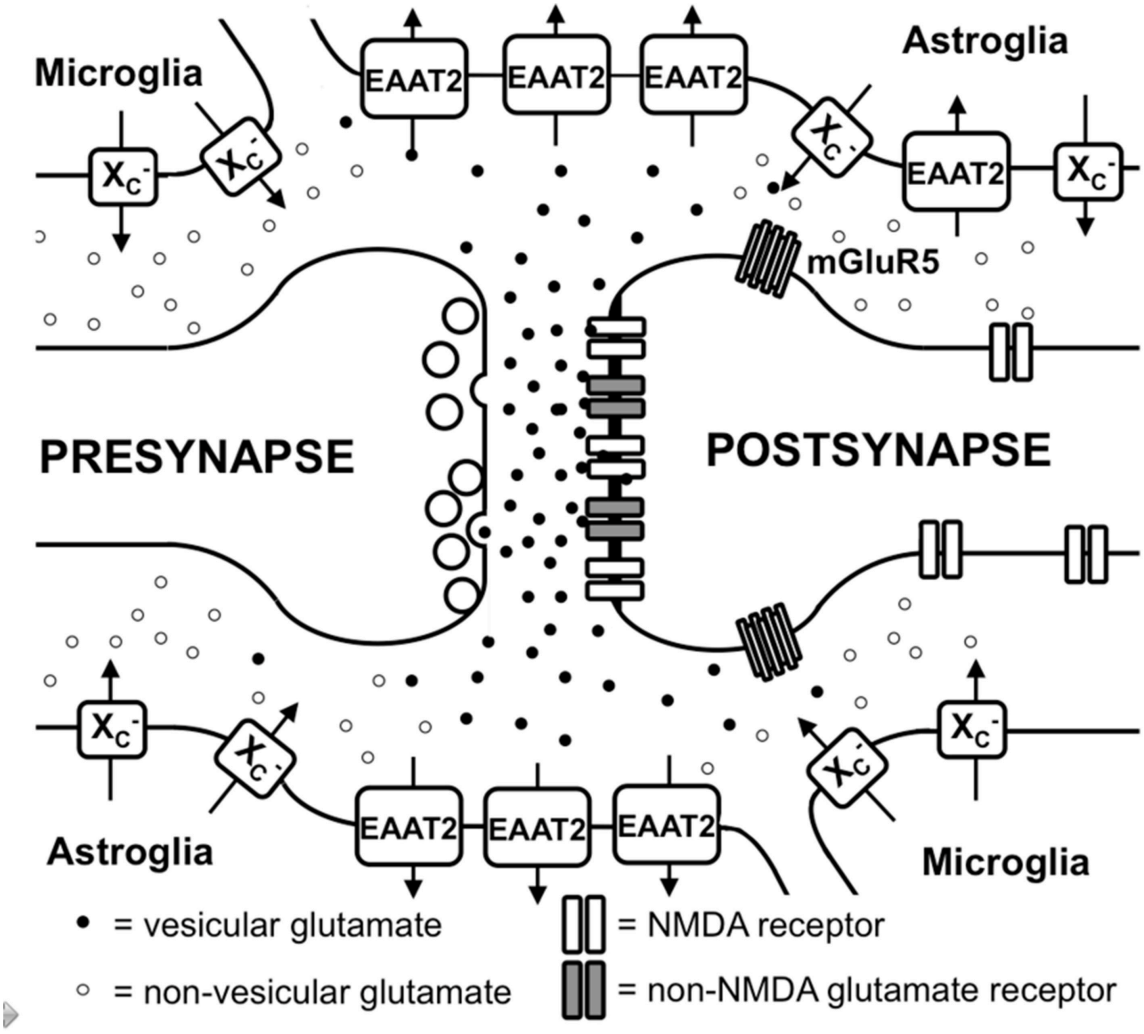
**Glutamate metabolism in the brain**. Vesicular glutamate is synaptically released and binds to ionotropic glutamate receptors at the postsynapse. Glutamate is rapidly taken up by astrocytic glutamate transporters, especially EAAT2. Spillover from the synaptic cleft can activate perisynaptic mGluR5. Migroglia and astrocytes non-vesicularly release glutamate into the extrasynaptic extracellular space via system x${}_{\text{c}}^{-}$ (x${}_{\text{c}}^{-}$) where it can activate extrasynaptic NMDA receptors.

L-glu is concentrated in synaptic vesicles in the presynaptic terminal by the action of vesicular glutamate transporters (vGLUT; Takamori, 2006). In addition, some of the L-glu in the vesicles might be generated by a vesicle-associated aspartate amino transferase from 2-oxoglutarate using L-aspartate (L-asp) as the amino group donor (Takeda et al., 2012). Upon depolarization of the presynaptic membrane, L-glu is released into the synaptic cleft and binds to ionotropic glutamate receptors (iGluRs) at the postsynaptic membrane (Figure 1). iGluRs are ligand-gated ion channels and include receptors of the α-amino-3-hydroxy-5-methyl-4-isoxazolepropionic acid (AMPA), kainate, and N-methyl-D-aspartic acid (NMDA) types (reviewed in Lodge, 2009). While AMPA and kainate receptors primarily mediate sodium influx, NMDA receptors have high calcium conductivity. Activation of NMDA receptors plays an important role in synaptic plasticity and learning (reviewed in Miyamoto, 2006). In contrast to the other iGluRs, the activity of NMDA receptors is inhibited by a so-called Mg^+2^ block at the regular membrane potential but the ion channel is readily de-blocked by membrane depolarization, which removes Mg^+2^ from the pore (Vargas-Caballero and Robinson, 2004). NMDA receptors are tetramers containing two NR1 subunits and two NR2 or NR3 subunits (Paoletti and Neyton, 2007).

In addition to iGluRs, eight isoforms of metabotropic glutamate receptors (mGluRs; Spooren et al., 2010) exist, which belong to the family of G-protein-coupled receptors and do not form ion channels but rather signal via various second messenger systems (reviewed in Spooren et al., 2010). L-glu-induced depolarization leads to a postsynaptic excitatory potential which facilitates the generation of an action potential at the axon hillock. The glutamatergic synapse is ensheathed by astrocytic processes that express high levels of excitatory amino acid transporters (EAATs; Chaudhry et al., 1995). Five different EAATs exist, EAAT1-5, of which EAAT1 and 2 are the primary astrocytic EAATs, whereas EAAT3 shows a predominantly neuronal expression (Zhou and Danbolt, 2013). About 90% of the L-glu transport is mediated by EAAT2 (aka GLT-1 in rodents). These transporters co-transport 2 or 3 molecules of Na^+^ and a proton with each molecule of L-glu (or L-asp) in conjunction with the counter-transport of a K^+^ ion (Zerangue and Kavanaugh, 1996). Thus, by using the electrochemical gradient of these ions across the plasma membrane as an energy source, the transporters are capable of effectively accumulating L-glu and L-asp in cells against their steep intra- to extracellular concentration gradients. This allows the brain to maintain a very low extracellular L-glu concentration in the low micromolar range (Baker et al., 2003; De Bundel et al., 2011). It is generally thought that L-glu taken up by astrocytes is converted to glutamine by the enzyme glutamine synthetase, the glutamine is then released, taken up by neurons and converted to L-glu and used once again for neurotransmission (Coulter and Eid, 2012).

### The role of extrasynaptic glutamate in the brain

Apart from the role of L-glu as the principal excitatory neurotransmitter released from glutamatergic presynapses as described above, it has become apparent that L-glu receptors outside the synaptic cleft also play an important role in brain physiology (Papouin and Oliet, 2014). In the cerebellum, it was demonstrated by analyzing AMPA receptor-mediated currents in Bergmann glia that synaptically released L-glu concentrations can reach extrasynaptic concentrations of up to 190 μM, while concentrations in the synaptic cleft exceed 1 mM (Dzubay and Jahr, 1999). Moreover, some mGluRs have been shown to exhibit a distinct localization in proximity to the postsynaptic density that would allow them to readily detect L-glu leaking from the synaptic cleft (Luján et al., 1997; Figure 1). However, in recent years, evidence has accumulated that iGluRs, especially of the NMDA type, are also present at extrasynaptic sites in the neuronal cell membrane (Papouin and Oliet, 2014). Using light and electron microscopy, Petralia et al. showed that extrasynaptic NMDA receptors cluster at distinct sites of close contact of the dendritic shaft with either axons, axon terminals, or astrocytic processes (Petralia et al., 2010). The proportion of extrasynaptic NMDA receptors was estimated to be as high as 36% of the dendritic NMDA receptor pool in rat hippocampal slices (Harris and Pettit, 2007). Although extrasynaptic NMDA receptors were associated with similar scaffolding proteins as synaptic NMDA receptors (Petralia et al., 2010), an *in vitro* study suggested that extrasynaptic and synaptic NMDA receptors may activate different downstream signaling pathways with contrasting results: suppression of CREB activity by extrasynaptic NMDA receptor activation but activation by synaptic NMDA receptors (Hardingham et al., 2002). Functionally, NMDA receptors localized extrasynaptically on dendritic shafts bind extrasynaptic L-glu and mediate Ca^2+^ influx upon relief of the Mg^+2^ block by dendrite depolarization upon backfiring of action potentials (Wu et al., 2012). Angulo et al. showed that L-glu release from astrocytes can activate so-called slow inward currents via extrasynaptic NMDAR receptors in CA1 neurons which thereby can be synchronized (Angulo et al., 2004). Thus, the mechanisms through which glial cells release L-glu as well as how the extrasynaptic L-glu concentrations are regulated are pivotal to understanding how the activity of extrasynaptic NMDA receptors are regulated.

Different mechanisms through which astrocytes can release L-glu have been proposed: vesicular L-glu release (Adak et al., 2000) and non-vesicular release via anion channels (Wang et al., 2013) and connexin hemichannels (Stehberg et al., 2012) as well as release via the cystine/glutamate antiporter system x${}_{\text{c}}^{-}$ (Massie et al., 2015). Data by Wang et al. strongly suggest that vesicular release from astrocytes plays a minor role, as the Ca^+2^-mediated release of L-glu was still present in astrocytes derived from dominant-negative SNARE mice (Wang et al., 2013) where vesicular release can be blocked by doxycycline withdrawal (Pascual et al., 2005). System x${}_{\text{c}}^{-}$ is a cystine/glutamate antiporter which belongs to the class of heterodimeric amino acid transporters, consisting of xCT as the specific subunit and 4F2hc as the promiscuous heavy chain (Sato et al., 1999). This transporter is expressed in the brain, especially in astroglial and microglial cells (Fogal et al., 2007; Mesci et al., 2015; Figure 1). The fact that extrasynaptic L-glu levels in different areas of the brain are downregulated by approximately 60–70% in xCT knock out mice (De Bundel et al., 2011; Massie et al., 2011) indicates that system x${}_{\text{c}}^{-}$ releases L-glu into the extrasynaptic space and demonstrates that this transporter is important in the regulation of extrasynaptic L-glu levels. This is further supported by the observation that when measured by *in vivo* microdialysis, the rise in extrasynaptic L-glu induced by EAAT inhibitors is neutralized by blocking system x${}_{\text{c}}^{-}$ while blocking neuronal vesicular L-glu release is ineffective (Baker et al., 2002; Melendez et al., 2005).

Taken together, glutamatergic neurotransmission not only occurs via classical excitatory synapses but also via extrasynaptic L-glu receptors (Figure 1). Moreover, the levels of extrasynaptic L-glu are determined, at least in part, by glial non-vesicular L-glu release (Figure 1). However, the regulation of extrasynaptic L-glu levels as well as its temporal-spatial dynamics and its impact on neuronal function, neurodegeneration, and behavior are far from being fully understood.

### Other molecules that are physiologically present in the brain and may activate glutamate receptors

Very early studies indicated that L-asp, like L-glu, has an excitatory action on neurons (Curtis et al., 1960). L-asp co-localizes with L-glu in the synaptic vesicles of asymmetric excitatory synapses (Gundersen et al., 1998). However, the total concentration in the brain (0.96–1.62 μmol/g wet weight) (Perry et al., 1971; Lefauconnier et al., 1976), the extracellular concentrations in the cortex as measured by microdialysis (1.62 μM for L-asp and 9.06 μM for L-glu) and its distribution as determined by immunohistochemistry (Gundersen et al., 1991) indicate that L-asp is less abundant that L-glu. However, L-asp is a potent agonist on NMDA receptors but not other iGluRs with an EC_50_ only eight-fold higher than that of L-glu (Patneau and Mayer, 1990). EAATs that play an important role in the uptake of vesicularly released L-glu in the CNS (Tanaka et al., 1997; Petr et al., 2015) also avidly take up L-asp (Arriza et al., 1994). Thus, L-asp is probably not as important as L-glu with respect to the total excitatory tone mediated by iGluRs but must not be forgotten in this context. In addition to its role as a neurotransmitter, as mentioned above, L-asp is also required as a substrate for aspartate amino transferase that converts 2-oxoglutarate to L-glu for transport into the synaptic vesicles of glutamatergic neurons (Takeda et al., 2012) and thus might indirectly increase L-glu release.

One feature that distinguishes NMDA receptors from other iGluRs is that activation of NMDA receptors requires the binding of a co-agonist to the glycine binding site of the receptor (Johnson and Ascher, 1987). Within the spinal cord and the retina, the source of glycine might be spillover from glycinergic inhibitory synapses (Ahmadi et al., 2003; Kalbaugh et al., 2009). However, in other parts of the brain with high NMDA receptor expression, like the hippocampal formation (Monyer et al., 1994), responses mediated by strychnine-sensitive glycine receptors are absent, at least in adult neurons, indicating the lack of glycinergic inhibitory neurotransmission (Ito and Cherubini, 1991). However, glycine is present in the extracellular fluid in the hippocampus at baseline levels of approximately 1.5 μM (Yamamoto et al., 2010), which is near the saturation of the glycine binding site of the NMDA receptor (Johnson and Ascher, 1987), but can be dynamically up- and down-regulated (Yamamoto et al., 2010). The source of extracellular glycine in the hippocampus might be neurons, which release glycine via the alanine-serine-cysteine amino acid transporter 1 (asc-1; Rosenberg et al., 2013). However, glycine release by astrocytes, which is stimulated by depolarization and kainate, has also been reported (Holopainen and Kontro, 1989).

Even very early studies of the NMDA receptor and its co-activation by glycine showed that D-amino acids, especially D-serine, are almost as effective as glycine (Kleckner and Dingledine, 1988). Only a few years later, it became apparent that D-serine is present in rat and human brains at approximately one third of the concentration of L-serine with an absolute concentration of more that 0.2 μmol/g brain tissue (Hashimoto et al., 1992, 1993). Using an antiserum specific for D-serine, Schell et al. demonstrated that D-serine in the brain is exclusively localized in astrocytes and that its distribution matches the expression of NMDA receptors (Schell et al., 1995). Moreover, the same authors showed that D-serine is released from cultured astrocytes upon exposure to L-glu or kainate (Schell et al., 1995). The abundance of D-serine is determined by both the degrading enzyme D-amino acid oxidase (DAO), which shows high expression in the hindbrain, where D-serine levels are low, and the synthetic enzyme serine racemase that generates D-serine from L-serine (Schell et al., 1995; Wolosker et al., 1999). D-Serine seems to be stored in cytoplasmic vesicles in astrocytes and is released by exocytosis (Mothet et al., 2005). Long-term potentiation depends on D-serine release from astrocytes in hippocampal slices, indicating that this amino acid indeed plays an important role in glutamatergic neurotransmission via NMDA receptors (Henneberger et al., 2010). Also in hippocampal slices, Papouin et al. determined, using D-serine and glycine degrading enzymes, that D-serine serves as a co-transmitter for synaptic NMDA receptors on CA1 neurons whereas glycine serves as the endogenous co-agonist for extrasynaptic NMDA receptors (Papouin et al., 2012). This, however, is not a general rule as synaptic NMDA receptors of dentate gyrus neurons use glycine instead of D-serine as the co-agonist (Le Bail et al., 2015).

Taken together, multilayered evidence suggests that not only L-asp acting as an agonist on NMDA receptors but also glycine as well as D-serine play important roles in glutamatergic neurotransmission in the brain. However, other molecules have also been proposed to be biologically relevant modulators of glutamatergic neurotransmission.

L-homocysteate (L-HCA) shares structural similarities with L-glu. This non-protein amino acid is an oxidation product of homocysteine (Frauscher et al., 1995) which is biosynthesized from methionine by the removal of its terminal methyl group and is an intermediate of the transsulfuration pathway through which methionine can be converted to cysteine via cystathionine (McBean, 2012). Early studies showed that this amino acid can induce calcium influx in cultured neurons as efficiently as L-glu (Berdichevsky et al., 1983). Indeed, L-HCA showed a high affinity for NMDA receptors as compared to other iGluRs in binding assays which correlated with its ability to induce NMDA receptor antagonist-inhibitable excitotoxicity and sodium influx (Pullan et al., 1987). In addition, L-HCA is able to activate mGluR5 as effectively as L-glu (Shi et al., 2003). L-HCA is present in the brain although the concentrations were found to be about 500-fold lower than those of L-glu and 100-fold lower than those of L-asp in different areas of the rat brain (Kilpatrick and Mozley, 1986). Upon potassium-induced stimulation, L-HCA release is induced from brain slice preparations as observed for L-asp and L-glu although the absolute release of HCA is about 50-fold lower (Do et al., 1986). Interestingly, HCA is a highly effective competitive inhibitor of cystine and L-glu uptake via the cystine/glutamate antiporter system x${}_{\text{c}}^{-}$ (Bannai and Ishii, 1982; Patel et al., 2004), the activity of which regulates the extracellular extrasynaptic L-glu concentrations in the brain (De Bundel et al., 2011). Thus, the effect of L-HCA on the activation of NMDA and other L-glu receptors (Yuzaki and Connor, 1999) might also depend on the L-HCA-induced release of L-glu via system x${}_{\text{c}}^{-}$. In summary, L-HCA may only play a limited role in the overall stimulatory input on L-glu receptors. However, this may change dramatically under some conditions, e.g., in patients with high-dose methotrexate therapy, an anticancer drug that, by inhibiting dihydrofolate reductase, inhibits the tetrahydrofolate-catalyzed recycling of methionine from homocysteine. Here, L-HCA concentrations of more than 100 μM have been documented in the cerebrospinal fluid whereas L-HCA was undetectable in control subjects (Quinn et al., 1997).

**Figure 2:**
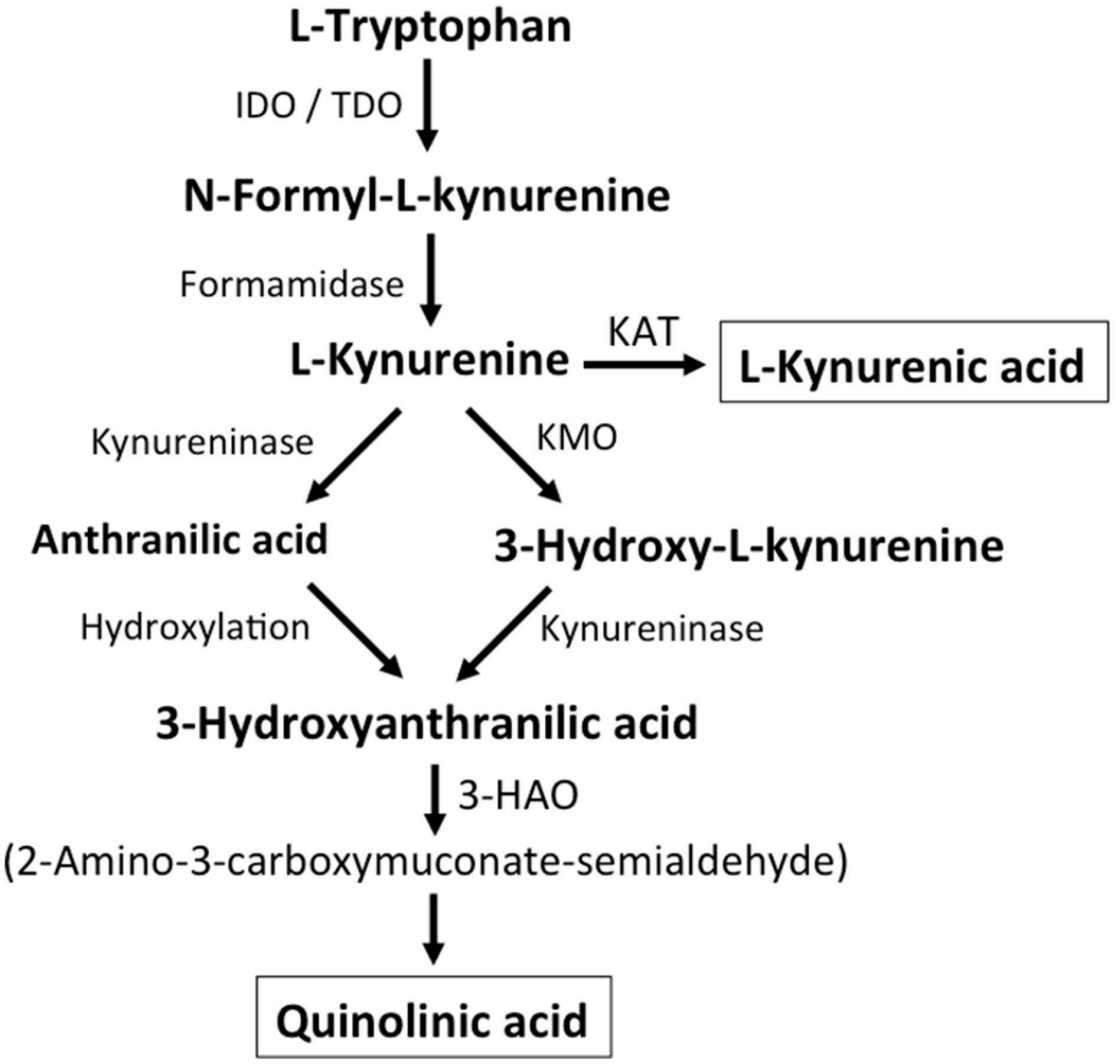
**Kynurenine metabolism**. By either indoleamine-2,3-dioxygenase (IDO) or tryptophan-2,3-dioxygenase (TDO), tryptophan is converted to N-formyl-L-kynurenine, which is metabolized to L-kynurenine by formaminidase. L-kynurenine may be metabolized to kynurenic acid by kynurenine aminotransferase (KAT), to 3-hydroxy-L-kynurenine by kynurenine-3-monooxygenase or to anthranilic acid by kynureninase. 3-Hydroxy-L-kynurenine and anthranilic acid can both be converted to 3-hydroxyanthranilic acid. 3-Hydroxyanthranilate oxidase (3-HAO) metabolizes 3-hydroxyanthranilic acid to 2-amino-3-carboxymuconate-semialdehyde which is non-enzymatically converted to quinolinic acid. Inhibition of KMO results in increased production of kynurenic acid while the generation of quinolinic acid is inhibited (modified from Vécsei et al., 2013).

Additional endogenous small molecules that are thought to interfere with L-glu signaling are several intermediates of tryptophan metabolism (Vécsei et al., 2013; Figure 2). Via the action of indoleamine 2,3-dioxygenase (IDO) or tryptophan 2,3-dioxygenase (TDO), tryptophan is converted to N-formyl-L-kynurenine, which is then converted to kynurenine (KYN) by formamidase. From here three pathways, two of which merge at a later step, lead to further metabolism. First, via the action of kynurenine aminotransferase (KAT), KYN is converted to kynurenic acid (KYNA). KYN also can be converted to 3-hydroxykynurenine (3HK) by kynurenine monooxygenase (KMO), which can then be used as a substrate by kynureninase for the synthesis of 3-hydroxyanthranilic acid (3HANA). In addition, using KYN as a substrate, kynureninase produces anthranilic acid (ANA), which by non-specific hydroxylation can also be converted to 3HANA. 3HANA finally serves a substrate for the generation of quinolinic acid (QUIN).

The tryptophan concentration in rat brain is about 25 nmol/g wet weight and thus about 400-fold less than L-glu and 100-fold less than L-asp (Gál and Sherman, 1978; Kilpatrick and Mozley, 1986). The reported brain concentrations of kynurenines are even lower with 0.4–1.6 nmol/g for QUIN (Wolfensberger et al., 1983), 0.01–0.07 nmol/ml for KYNA (Moroni et al., 1988), and 0.016 nmol/g for 3HANA (Baran and Schwarcz, 1990). About 40% of brain KYN is locally synthesized (Gál and Sherman, 1978). The different metabolites of tryptophan show differential binding to plasma proteins and their transport via the blood-brain barrier is very different. KYN and 3HK are transported via the large neutral amino acid carrier system L (Fukui et al., 1991). Other kynurenines seem to enter the brain by passive diffusion. In addition, ANA, 3HANA and especially KYNA avidly bind to serum proteins, which limit their diffusibility across the blood-brain barrier.

As early as 1982, it was found that QUIN, when ionophoretically applied to rat cortex, induced neuronal firing that was inhibited by an NMDA receptor antagonist (Birley et al., 1982), indicating that QUIN may act as an NMDA receptor agonist. However, the EC_50_ for QUIN to activate NMDA receptor currents has been shown to be approximately 1000-fold higher than the EC_50_ of L-glu (Patneau and Mayer, 1990). Of note, intracerebral injection of QUIN has been shown to induce ultrastructural, neurochemical, and behavioral changes very similar to those induced by NMDA receptor agonists (Schwarcz et al., 1983). However, the fact that QUIN concentrations are about 5000- to 15,000-fold lower than cerebral L-glu concentrations makes it highly unlikely that modulation of NMDA receptor signaling by QUIN plays a significant role. KYNA was reported to act as an NMDA receptor antagonist (Ganong et al., 1983; Ganong and Cotman, 1986). However, although infusion with the KMO inhibitor Ro 61-8048 increased cerebral extracellular KYNA concentrations 10-fold, this did not lead to an inhibition of NMDA-mediated neuronal depolarization, a finding that challenges the notion that KYNA at near physiological levels directly modulates NMDA receptors (Urenjak and Obrenovitch, 2000). In contrast, increased KYNA in the brain induced by the KMO inhibitor JM6 decreased the extracellular cerebral L-glu concentration (Zwilling et al., 2011). In addition, KYNA levels in the extracellular cerebral fluid highly correlated with L-glu levels indicating that even at physiological or near physiological levels KYNA modulates L-glu metabolism (Zwilling et al., 2011). Both activation of the G-protein-coupled receptor GPR35 (Berlinguer-Palmini et al., 2013) and inhibition of presynaptic α7 nicotinic acetylcholine receptors have been implicated in the KYNA-induced reduction in L-glu release (Carpenedo et al., 2001). To summarize, although L-HCA and QUIN are present in the brain, their low concentrations speak against them having prominent roles in regulating glutamatergic neurotransmission. In contrast, although the pathways still have to be defined in more detail, evidence supports the view that KYNA can modulate L-glu release and thereby L-glu levels and neurotransmission.

## The concepts of acute and chronic glutamate toxicity

The term excitotoxicity was first used by Olney (1986) to describe the ability of L-glu, as well as structurally related amino acids, to kill nerve cells, a process that has been proposed to take place not only in acute but also chronic diseases of the central nervous system (for reviews Choi, 1988; Meldrum and Garthwaite, 1990). Excitotoxicity results from the excessive activation of iGluRs and leads to a characteristic loss of post-synaptic structures including dendrites and cell bodies. There is a significant level of variation in the sensitivity of different nerve cells to the various iGluR agonists which is related to both the specific receptors expressed on the nerve cells as well as their distinct metabolisms. Moreover, the susceptibility of neurons to excitotoxicity can change tremendously with age. Acute excitotoxic nerve cell death is thought to occur in response to a variety of severe insults including cerebral ischemia, traumatic brain injury (TBI), hypoglycemia, and status epilepticus (Meldrum and Garthwaite, 1990; Meldrum, 1994). But what about neurodegenerative diseases where nerve cell death occurs over an extended period of time? Does chronic excitotoxicity also exist? In other words, could exposure of nerve cells to low but above normal concentrations of L-glu (or glutamatergic neurotransmission as a sum of the input via the various molecules involved as discussed above) over an extended period of time also eventually lead to nerve cell death?

Excitotoxicity was originally studied in animals but in order to understand the mechanisms underlying this process, cell culture models were developed (for review Choi, 1988). The basic cell culture model of acute excitotoxicity involves treating primary neurons in culture with L-glu or a specific iGluR agonist for a short time period (min) (e.g., Choi, 1992; Schubert and Piasecki, 2001) and then assessing downstream events at the time point that is most relevant for the study. For example, cell death is often determined after 24 h. While these types of studies have been shown to be very useful for understanding the pathways involved in acute excitotoxicity, it has proven much more difficult to study chronic excitotoxicity in culture partly because it is not entirely clear how to define “chronic” in the context of cell culture. Does chronic mean a low dose given for 24 h rather than a higher dose given for 5–10 min or is it more complicated than that? In one of the few studies that attempted to develop a model of chronic excitotoxicity (Ha et al., 2009), it was shown that it is indeed more complicated with acute and chronic excitotoxicity appearing to be distinct processes. In this study, the authors used pure cultures of primary cortical neurons prepared from day 14 mouse embryos and treated them after 7 and 14 days in culture (DIV; Ha et al., 2009). For chronic excitotoxicity, the neurons were exposed to L-glu or NMDA for 24 h and for acute excitotoxicity for 10 min. In both cases, cell death was measured after 24 h. Surprisingly, the EC_50_s for the toxicity of L-glu were lower for acute toxicity, especially in the 7 DIV cultures, as compared to the EC_50_s for chronic toxicity. In addition, it was found that a high cell culture density increased the sensitivity of the cells to acute but not chronic excitotoxicity. Further studies suggested that the lower sensitivity of the neurons to L-glu in the chronic excitotoxicity paradigm was due to the activation of mGluR1, consistent with earlier data on the neuroprotective effects of mGluR1 activation (Sagara and Schubert, 1998).

An alternative approach to studying chronic glutamate toxicity utilized organotypic spinal cord cultures in combination with L-glu uptake inhibitors (Rothstein et al., 1993). These spinal cord cultures, which were prepared from 8-day-old rat pups, can be maintained in culture for up to 3 months. Chronic inhibition of L-glu uptake with two different uptake inhibitors resulted in a persistent elevation of L-glu in the cell culture medium and time- and concentration-dependent motor neuron cell death. The highest concentration of uptake inhibitor increased extracellular L-glu levels at least 25-fold and began to kill the cells within 1 week whereas a five-fold lower concentration raised extracellular L-glu levels eight-fold and cell death only began after 2–3 weeks of treatment. The toxicity was blocked by non-NMDA but not NMDA receptor antagonists as well as by inhibitors of L-glu synthesis or release. These experiments suggest that even moderately increased L-glu concentrations can induce toxicity.

*In vivo* approaches to studying chronic excitotoxicity have mainly relied on an approach analogous to that used with the spinal cord cultures. In the majority of these studies, one or more EAATs were transiently or permanently genetically eliminated and the effects on brain function examined. In the first of these studies that used rats (Rothstein et al., 1996), chronic intraventricular administration of antisense RNA was used to eliminate each of the three main EAATs (EAAT1, EAAT2, and EAAT3). The loss of either of the glial L-glu transporters (EAAT1 and EAAT2) but not the neuronal transporter (EAAT3) resulted in large increases in extracellular L-glu concentrations in the striatum after 7 days as determined by microdialysis (EAAT2, 32-fold increase; EAAT1, 13-fold increase). Treatment with either the EAAT1 or EAAT2 antisense oligonucleotides caused a progressive motor impairment whereas the EAAT3 antisense oligonucleotide mainly produced epilepsy. The loss of any of the three transporters produced clear evidence of neuronal damage in the striatum and hippocampus after 7 days of treatment although the effects of the EAAT1 and EAAT2 antisense oligonucleotides were much more dramatic, consistent with the large increases in extracellular L-glu brought about by treatment with these antisense oligonucleotides.

Quite different results were obtained with homozygous mice deficient in EAAT2 (Tanaka et al., 1997) or EAAT1 (Watase et al., 1998). Mice deficient in EAAT2 displayed spontaneous and generally lethal seizures with 50% dead by 6 weeks of age (Tanaka et al., 1997). Approximately 30% of the mice showed selective neuronal degeneration in the hippocampal CA1 region at 4–8 weeks of age. L-glu levels in the CA1 region of the hippocampus measured by microdialysis were ~three-fold higher in the mutant mice as compared with the wild type mice (Mitani and Tanaka, 2003). In contrast, heterozygous EAAT2 knock-out mice have a normal lifespan and do not show hippocampal CA1 atrophy (Kiryk et al., 2008). However, they display some behavioral abnormalities suggestive of mild glutaminergic hyperactivity. While mice deficient in EAAT1, which is highly expressed in cerebellar astrocytes, did not show changes in cerebellar structure or obvious symptoms of cerebellar impairment such as ataxic gait, they were unable to adapt to more challenging motor tasks such as quickly running on the rotorod (Watase et al., 1998). Taken together, these results suggest that disruptions in glutamatergic homeostasis have a much greater impact when they occur in the adult animal rather than when they are present from conception.

Tuberous sclerosis complex (TSC) is a multi-system genetic disease caused by mutation of either the *TSC1* or *TSC2* genes and characterized by severe neurological symptoms. Mice with inactivation of the *TSC1* gene in glia have a >75% decrease in the expression and function of EAAT1 and EAAT2 and develop seizures (Zeng et al., 2007). At 4 weeks of age, prior to the development of seizures in these mice, there was an ~50% increase in extracellular L-glu in the hippocampus of these mutant mice, as determined by microdialysis, which correlated with increases in markers of cell death in neurons in both the hippocampus and cortex. Using hippocampal slices from 2 to 4 week old mice, impairments in long-term potentiation were identified, which translated into functional deficits when juvenile mice were tested for spatial and contextual memory in the Morris water maze and fear conditioning assays, respectively.

In most of the studies described above, there was a large increase in extracellular L-glu which, when examined, led, in most cases to negative impacts on the function of specific neuronal populations. To determine the long-term effects of more moderate increases in extracellular glutamate, Bao et al. (2009) created transgenic (Tg) mice with extra copies of the gene for *Glud1* specifically in neurons. The GLUD1 protein is a mitochondrial enzyme that converts L-glu to 2-oxoglutarate. Mitochondrial 2-oxoglutarate is transported to the cytoplasm of nerve terminals where it is converted back into L-glu and stored in synaptic vesicles thereby contributing to the pool of synaptically releasable L-glu (Palaiologos et al., 1988; Takeda et al., 2012). Nine month old Glud1 Tg mice showed an ~10% increase in L-glu in the hippocampus and striatum relative to wild type mice as determined using magnetic resonance spectroscopy. In addition, evoked L-glu release in the striatum was increased by ~50%. At 12–20 months of age, the Glud1 Tg mice showed significant decreases in the numbers of neurons in the CA1 region of the hippocampus and granule cell layer of the dentate gyrus as well as an age-dependent loss of both dendrites and dendritic spines in the hippocampus. There was also a decrease in long-term potentiation following high frequency stimulation in hippocampal slices from the Tg mice as compared to the wild type mice. However, whether these alterations resulted in behavioral impairments was not examined. Analysis of the transcriptome of the Glud1 Tg mice as compared to wild type mice suggested that long-term moderate increases in extracellular L-glu result in both accelerated aging at the level of gene expression coupled with compensatory responses that protected against stress and/or promoted recovery (Wang et al., 2010).

In summary, increases in extracellular L-glu *in vivo* can affect nerve cell survival and brain function. The consequences seem to be highly dependent on the degree of L-glu increase but even a 10% increase appears to affect nerve cell structure and survival particularly in the context of aging suggesting that chronic excitotoxicity may be particularly relevant to age-related neurodegenerative diseases.

Plant-based toxins have provided additional evidence for chronic excitotoxicity. These include domoic acid, the cause of amnesiac shellfish poisoning, from the alga *Pseudo-nitzschia* (Grant et al., 2010), β-*N*-oxalylamino-L-alanine (BOAA), the cause of lathyrism, from the plant *Lathyrus sativus* (Ludolph and Spencer, 1995) and possibly β-*N*-methylamino-L-alanine (BMAA), a postulated cause of Guamian amyotrophic lateral sclerosis/Parkinson-dementia complex (ALS/PDC), from the cycad *Cycas circinalis* (Karamyan and Speth, 2008). Based on evidence from human, and in some cases animal, exposures that resulted in significant neurological damage, further studies on these toxins showed that they all act on L-glu receptors and induce both acute and chronic neurotoxicity.

Domoic acid binds with high affinity to AMPA-, kainate-, and NMDA-type iGluRs (Qiu et al., 2006). Both acute, high-dose toxicity, which can include seizures and death as well as lower dose, chronic toxicity of domoic acid have been described in both humans and animals that consumed shellfish and other sea life that eat the alga and concentrate the toxin (Grant et al., 2010). Domoic acid-related neuropathology occurs mainly in the hippocampus and results in memory deficits which may be permanent (Grant et al., 2010). Studies in animals suggest that domoic acid toxicity can be progressive over time with a significant period of good health after exposure before the manifestation of delayed injury (Grant et al., 2010). Moreover, the toxicity appears to be exacerbated by increasing age (Qiu et al., 2006).

Lathyrism is a neurological condition that develops only after several months of ingestion of the grass pea and only when it constitutes at least 30% of a nutritionally-poor diet (Paleacu et al., 1999). BOAA binds preferentially to non-NMDA-type iGluR. In contrast to domoic acid, BOAA specifically and selectively targets the upper motor neurons resulting in spastic paraparesis (Ludolph and Spencer, 1995). Lathyrism is not associated with any cognitive problems (Paleacu et al., 1999).

BMAA, which is closely related to BOAA, binds to both NMDA-type and non-NMDA-type iGluRs (Lobner et al., 2007). In addition, it can both inhibit cystine uptake into cells via system x${}_{\text{c}}^{-}$ thereby promoting oxidative stress and, as it is a substrate inhibitor, promote L-glu release from cells via the same transporter, which can enhance excitotoxicity through activation of mGluR5 (Liu et al., 2009). While the evidence that it could be responsible for Guanamian ALS/PDC is still debatable after almost 50 years (Karamyan and Speth, 2008), oral administration of BMAA to macaques dose-dependently caused a motor system disorder with involvement of both the upper and lower motor neurons as well as the extrapyramidal system after 2–12 weeks of treatment (Spencer et al., 1987). Histological examination showed that the motor cortex was most affected followed by the spinal cord and then the substantia nigra, which was mostly spared. While these symptoms resembled the early stages of Guanamian ALS/PDC, the severe cognitive deficits associated with the disorder were not seen. However, this could be related to the relatively short treatment period, the relatively young age of the macaques and/or the nutritional adequacy of their diet.

In summary, several toxins that bind to iGluRs and have been shown to induce excitotoxicity in cell culture can cause slowly developing neurological problems in both humans and animals. Interestingly, each toxin appears to target a specific type of neuron, an effect that might be related to both the pharmacokinetic and ADME properties of the toxins, which have not yet been studied to any great extent. However, the data from these toxins supports the idea that chronic excitotoxicity exists in humans and could play a role in multiple neurological disorders.

Since iGluRs are found both in the synapse as well in extra-synaptic locations, there has been a great deal of effort devoted to determining if the location of the receptors impacts the toxicity of glutamate and related molecules. An influential study using primary neuronal cultures (Hardingham et al., 2002) suggested that synaptic and extrasynaptic NMDA receptors have counteracting effects on cell fate with nerve cell death being mainly mediated by extrasynaptic NMDA receptors. However, these results have not been reproduced in brain slices or *in vivo* (reviewed in Papouin and Oliet, 2014). Furthermore, several more recent studies using the same primary neuronal culture preparation protocol as the earlier study found either no difference between synaptic and extrasynaptic NMDA receptors in promoting excitotoxicity (reviewed in Papouin and Oliet, 2014) or found that both receptors were required for cell death (Zhou et al., 2013). Moreover, a number of studies that supported the idea that extrasynaptic NMDA receptors promote excitotoxicity relied on the NMDA receptor inhibitor memantine which was originally thought to specifically act on extrasynaptic NMDA receptors (Xia et al., 2010). However, more recent studies have shown that memantine can inhibit both synaptic and extrasynaptic NMDA receptors (Emnett et al., 2013). Together these results strongly suggest that both synaptic and extrasynaptic NMDA receptors can contribute to excitotoxicity but that the precise contribution of each may depend on the experimental and/or pathological conditions.

## Evidence for glutamate dysregulation and excitotoxicity in different neurodegenerative diseases

### Excitotoxicity in acute diseases of the CNS

As mentioned above, excitotoxicity was initially defined as an acute insult to nerve cells that leads to cell death by excessive activation of iGluRs. Acute excitotoxicity is known to play an important role in specific CNS disorders including cerebral ischemia, TBI, and status epilepticus. However, the mechanisms underlying acute excitotoxicity differ slightly among these different disorders as described below.

During brain ischemia, the initiation of L-glu- (or L-asp-) mediated excitotoxicity occurs within minutes due to the rapid increase in extracellular cerebral L-glu (and L-asp; reviewed in Dirnagl et al., 1999). The sudden loss of the energy supply due to the shut down of blood flow to the brain leads to a breakdown of the neuronal and astroglial membrane potentials as the maintenance of these is energy-dependent. In neurons, the subsequent membrane depolarization leads to vesicular L-glu release. In addition, energy depletion and disruption of ionic homeostasis inhibits EAAT activity in astrocytes and may even induce a reversal in their action thereby leading to non-vesicular L-glu and L-asp release. The release of L-glu/L-asp (Graham et al., 1990) from these different sources leads to excitotoxicity, mostly by over-activation of iGluRs of the NMDA type as shown by the efficacy of NMDA antagonists in the acute phase in animal models of transient cerebral ischemia (Park et al., 1988; Bielenberg and Beck, 1991; Katsuta et al., 1995).

In TBI, the mechanical tissue damage and disruption of the blood-brain barrier is the initiator of acute secondary neurodegeneration, which, in addition to neuroinflammation and oxidative stress, is mediated by L-glu release from intracellular compartments and thus by acute excitotoxicity (reviewed in Freire, 2012). Accordingly, acute administration of the NMDA antagonist MK801 following TBI ameliorates neuronal loss and long-term behavioral abnormalities (Sönmez et al., 2015).

In status epilepticus, ongoing synchronized activity of excitatory neuronal networks with concurrent breakdown of inhibitory mechanisms is the primary source of increased L-glu (and L-asp) release. As the intensity of synchronous activity is dependent on the integration of a nerve cell into a specific neuronal network and the ability of a nerve cell to withstand excessive glutamatergic input depends, among other properties, on the expression pattern of iGluRs, a rather restricted and maturation-dependent degeneration of neuronal populations is induced by prolonged epileptic seizures (Sankar et al., 1998). The relevance of excitotoxicity in status epilepticus is demonstrated by the fact that NMDA antagonists like ketamine reduce neuronal loss (Loss et al., 2012).

### Chronic excitotoxicity during progressive long-term neurodegeneration

As compared to acute insults to the CNS, in chronic neurodegenerative diseases the situation is much more complex. First, although compromised mitochondrial function has been repeatedly described in several neurodegenerative diseases (reviewed in Johri and Beal, 2012), the resulting impairments in energy supply are not nearly as severe as the energy failure in ischemic stroke. Thus, if excitotoxicity contributes to neurodegeneration, a very different time course of chronic excitotoxicity has to be assumed. In the following paragraphs, we will summarize what is known about the different pathways that might contribute to excitotoxicity in neurodegenerative diseases. We will focus on amyotrophic lateral sclerosis (ALS), Alzheimer's disease (AD) and Huntington's disease (HD) as important examples with sufficiently validated animal models.

#### Amyotrophic lateral sclerosis

ALS is a neurodegenerative disease where the degeneration of motor neurons dominates the clinical presentation and the course of the disease, which is fatal within a few years from onset. It is hypothesized that L-glu excitotoxicity plays a role in the motor neuron death in ALS as these cells express high levels of calcium-permeable AMPA receptors (Carriedo et al., 1996) and low levels of calcium binding proteins (Leal and Gomes, 2015). In contrast to the application of AMPA and kainate, as well as L-HCA, to the lumbar spinal cord of rats, treatment with NMDA spared motor neurons, indicating that NMDA excitotoxicity might not play a prominent role in ALS (Ikonomidou et al., 1996). However, NMDA receptor-mediated excitotoxicity in motor neurons was documented in chick embryo organotypic slice cultures (Brunet et al., 2009). Electrophysiological studies indicated that there is a transient hyperexcitability of motor neurons in the presymptomatic stage of ALS in mice transgenic for the G93A mutation of human SOD1 that is associated with hereditary ALS (Fuchs et al., 2013). In addition, a cortical hyperexcitability has been documented in familial and sporadic ALS patients that precedes the onset of clinical symptoms in familial ALS mutation carriers (Vucic et al., 2008). Finally, the only approved drug to treat ALS, which increases survival by 2–3 months (Miller et al., 2012), acts as an inhibitor of NMDA and kainate receptors (Debono et al., 1993) as well as rapidly upregulating EAAT activity in synaptosomes (Azbill et al., 2000).

In autopsied spinal cord from patients with ALS, several groups reported a pronounced reduction of EAAT2 but not EAAT1 protein expression in the gray matter in areas with significant motor neuron loss (Sasaki et al., 2000). In addition, both L-glu uptake as well as EAAT2 immunoreactivity, as analyzed by Western blotting, were shown to be quantitatively reduced in postmortem tissue of ALS patients especially in the spinal cord, the tissue that is most affected by the disease (Rothstein et al., 1992; Lin et al., 1998). Moreover, it was reported that as a potential consequence of EAAT2 downregulation, L-glu levels are increased in the CSF in patients with ALS (Rothstein et al., 1990). However, this finding could not be replicated by others (Perry et al., 1990).

The downregulation of EAAT2 in human ALS is recapitulated in several animal models of ALS including transgenic mice expressing human SOD1 containing the G93A mutation that causes hereditary ALS (Bendotti et al., 2001) or transgenic rats expressing the same mutation (Howland et al., 2002). Interestingly, whereas Bendotti found a late decrease in EAAT2 expression at the time when the mice have already become symptomatic (Bendotti et al., 2001), Howland et al. reported changes in EAAT2 expression in the presymptomatic stage (Howland et al., 2002). The β-lactam antibiotic ceftriaxone (Cef) induces EAAT2 in cultured murine spinal cord slices (Rothstein et al., 2005) and in neuron/astrocyte co-cultures (Lewerenz et al., 2009). It also induced EAAT2 expression in the spinal cords of wild-type and mutant G93A mSOD1 Tg mice, which was associated with a decrease in motor neuron loss, weight loss, and other ALS-like symptoms and an increase in survival (Rothstein et al., 2005), compatible with the idea that EAAT2 loss contributes to chronic excitotoxicity in this mouse model. Just recently, a prominent reduction in EAAT2 immunoreactivity was reported in an independent rodent model for ALS, rats expressing ALS-inducing mutant TAR DNA binding protein 43 in astrocytes only (Tong et al., 2013). Interestingly, Alexander et al. found that, when measured by microdialysis, the extracellular L-glu and L-asp concentrations are increased and the L-glu clearance capacity is decreased in the cerebral cortex of G93A mSOD1 Tg mice although this area does not show overt pathology nor downregulation of EAAT1 and 2 when analyzed by Western blotting (Alexander et al., 2000).

Taken together these publications support the view that there is a downregulation of EAAT2 in both human ALS patients and animal models of ALS. However, while some animal studies suggest that EAAT2 downregulation occurs prior to motor neuron loss, others are compatible with the hypothesis that the downregulation of EAAT2, the astroglial expression of which depends on the presence of neurons (Morel et al., 2013), is a consequence of neuronal degeneration.

Whereas, EAATs decrease extracellular L-glu, extracellular cerebral L-glu is upregulated in different brain regions by the cystine/glutamate antiporter system x${}_{\text{c}}^{-}$ (De Bundel et al., 2011; Massie et al., 2011). xCT, the specific subunit of system x${}_{\text{c}}^{-}$, was reported to be differentially regulated in mouse models of ALS. Albano et al. (2013) reported that the uptake of radiolabelled cystine was upregulated in spinal cord slices of presymptomatic G93A mSOD1 Tg mice at the age of 70 days but not at 55 or 100 days and not in symptomatic 130 day-old mice and confirmed that the upregulation of cystine uptake at day 70 was due to system x${}_{\text{c}}^{-}$ activity using the system x${}_{\text{c}}^{-}$ inhibitor sulfasalazine (SSZ). It has to be kept in mind, however, that cystine can also be transported by EAATs (Hayes et al., 2005). Thus, as data about the SSZ-sensitivity of the cystine uptake were not presented for days 100 and 130, the lack of differential cystine uptake found in this study at the older ages might rather be a consequence of decreased EAAT activity. In contrast, Mesci et al. (2015) using rtPCR, demonstrated a robust increase in xCT mRNA levels in G37R mSOD1 Tg mice upon the onset of symptoms, which was further increased as symptoms progressed (Mesci et al., 2015). Moreover, it was demonstrated that xCT was predominantly expressed in spinal cord microglia. When acutely purified from spinal cord, microglia already showed xCT mRNA upregulation in the presymptomatic phase. Taken together, these findings indicate that system x${}_{\text{c}}^{-}$ is upregulated in animal models of ALS. However, evidence is lacking about whether this is also true for human cases of ALS. Nevertheless, Mesci demonstrated that the mRNA levels of CD68, a marker of microglial activation, correlated with xCT mRNA expression in postmortem spinal cord tissue of patients with ALS, suggesting that neuroinflammation in humans is associated with xCT upregulation (Mesci et al., 2015).

Beyond the dysregulation of L-glu and L-asp levels by EAAT downregulation or system x${}_{\text{c}}^{-}$ upregulation, pathways that indirectly modulate glutamatergic neurotransmission have also been proposed to be involved in motor neuron degeneration in ALS. D-Serine levels have been shown to be progressively increased in the spinal cord of G93A mSOD1 Tg mice (Sasabe et al., 2007, 2012). Beginning at disease onset and continuing during the course of the symptomatic phase, D-serine augments NMDA excitotoxicity in motor neurons (Sasabe et al., 2007, 2012). The upregulation of D-serine in the spinal cord was replicated by others (Thompson et al., 2012). Downregulation of the D-serine metabolizing enzyme DAO in the reticulospinal tract was identified as the main mechanism for D-serine upregulation in the spinal cord in ALS mice (Sasabe et al., 2012). In addition, genetic inactivation of DAO in mice leads to motor neuron degeneration (Sasabe et al., 2012) and a deficiency in the D-serine generating enzyme serine racemase prolonged survival in G93A mSOD1 Tg mice although it paradoxically hastened disease onset (Thompson et al., 2012). A heterozygous mutation of DAO has been shown to segregate with the ALS phenotype in a large family with hereditary ALS (Mitchell et al., 2010). However, this remains the only family identified so far where a DAO mutation may be linked to the disease (Millecamps et al., 2010).

Regarding the other amino acid co-agonist of the NMDA receptor, glycine, an increase in the CSF levels in patients with ALS was reported by one group (de Belleroche et al., 1984) but could not be replicated by others (Perry et al., 1990; Rothstein et al., 1990). Ilzecka reported that KYNA levels are upregulated in the CSF of bulbar ALS patients and those in end stage disease (Ilzecka et al., 2003). Independently, it was described that tryptophan and KYN levels are increased in the CSF from ALS patients as compared to controls (Chen et al., 2010). In addition, IDO was shown to be expressed in spinal cord microglia and neurons from patients with ALS, indicating that microglial activation could increase the conversion of tryptophan to KYN in ALS.

**Figure 3:**
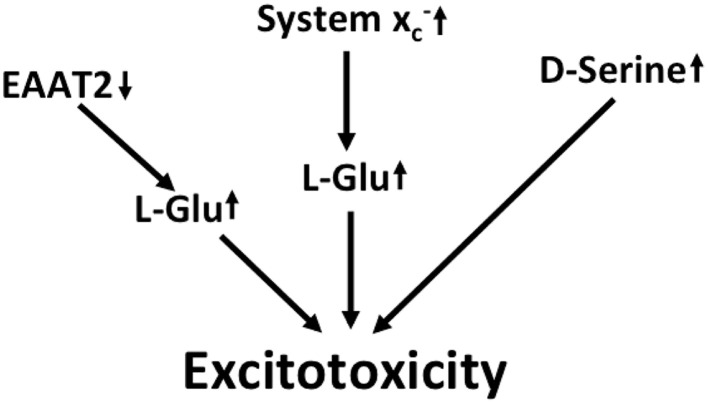
**Potential mechanisms that lead to excitotoxicity in ALS**. EAAT2 downregulation and upregulation of system x${}_{\text{c}}^{-}$ cause increased activation of glutamate receptors. Moreover, signaling via NMDA receptors is potentiated by the upregulation of the co-activator D-serine.

In summary, multilayered evidence suggests that increased glutamatergic neurotransmission is present in ALS and might contribute to neurodegeneration (Figure 3). Downregulation of EAAT2 in astrocytes and upregulation of system x${}_{\text{c}}^{-}$ in the context of microglial activation has been repeatedly documented. Increased co-activation of NMDA receptors by D-serine might also play a role in glutamatergic dysregulation. In addition, the kynurenine pathway seems to be activated in ALS, most likely in the setting of neuroinflammation.

#### Alzheimer's disease

AD is the leading cause of dementia in the aging population. Neuropathologically, AD is defined by neurodegeneration with the presence of extracellular senile plaques consisting of β amyloid (Aβ) and intraneuronal neurofibrillary tangles consisting of aggregated tau (Grundke-Iqbal et al., 1986; Ingelsson et al., 2004), which first appear in the hippocampus and then spread as the disease progresses. Prominent microglial activation is also a hallmark of AD. Hereditary forms of AD are caused by mutations in the Aβ precursor protein, AβPP, or in the presenilins, which are part of the multi-protein complex involved in Aβ generation (Ringman et al., 2014). The pathophysiology of AD is complex and many pathways are involved in the synaptic and cellular degeneration in AD including abnormalies in signaling pathways via glycogen synthase kinase-3 beta or mitogen-activated protein kinases, cell cycle re-enty (reviewed by Majd et al., 2015), oxidative stress (reviewed by Niedzielska et al., 2015), or decreased transport of trophic factors and mitochondrial dysregulation (reviewed by Overk and Masliah, 2014). However, multiple lines of evidence suggest that L-glu dysregulation also plays a role in AD.

Grilli et al. showed that primary neurons from transgenic mice overexpressing mutant presenilin are more sensitive to excitotoxic stimuli *in vitro* (Grilli et al., 2000). *In vitro*, aggregated Aβ enhances both NMDA and kainate receptor-mediated L-glu toxicity, most likely by interfering with neuronal calcium homeostasis (Mattson et al., 1992). Others have shown that Aβ can increase neuronal excitability by impairing the ability of glycogen synthase kinase 3β inhibition to reduce NMDA receptor-mediated currents (Deng et al., 2014). Moreover, soluble Aβ oligomers were found to induce L-glu release from astrocytes eventually leading to dendritic spine loss via over-activation of extrasynaptic NMDA receptors (Talantova et al., 2013). Extracellular L-glu concentrations were found to be increased in a triple transgenic mouse model of AD, in which a 3-month treatment with the NMDA receptor inhibitor NitroMemantine rescued synapse loss (Talantova et al., 2013).

A number of mouse studies have shown impacts of AD-like pathology on EAAT expression and/or function. In acute hippocampal slice preparations, Aβ was reported to inhibit the clearance of synaptically released L-glu by decreasing membrane insertion of EAAT2, an effect probably mediated by oxidative stress (Scimemi et al., 2013). In aged AβPP23 mice, Schallier et al. observed the downregulation of EAAT2 expression in the frontal cortex and hippocampus, which in the frontal cortex was associated with an increase in xCT expression (Schallier et al., 2011). These changes were associated with a strong tendency toward increased extracellular L-glu levels as measured by microdialysis (Schallier et al., 2011). In triple transgenic AD mice expressing the amyloid precursor protein mutations K670N and M671L, the presenilin 1 mutation M146V and the tau P301L mutation (Oddo et al., 2003; Zumkehr et al., 2015), a strong and age-dependent reduction of EAAT2 expression was found (Zumkehr et al., 2015). Restoration of EAAT2 activity in these AD mice following treatment with the β-lactam antibiotic Cef was associated with a decrease in cognitive impairment as well as reduced tau pathology (Zumkehr et al., 2015). In human AD brains, decreased expression of EAAT2 protein as well as a decrease in EAAT activity was described (Li et al., 1997). However, this finding could not be replicated by others (Beckstrøm et al., 1999). Of note, on the transcriptome level, Scott et al. found exon-skipping splice variants of EAAT2 that decrease glutamate transport activity to be upregulated in human AD brains (Scott et al., 2011). In the CSF, some groups found an increase in glutamate concentrations (Pomara et al., 1992; Csernansky et al., 1996; Jimenez-Jimenez et al., 1998; Kaiser et al., 2010) in AD patients, whereas others found no change or even decreased levels (Basun et al., 1990; Martinez et al., 1993; Kuiper et al., 2000).

*In vitro*, Aβ induces L-glu release from primary microglia via upregulation of system x${}_{\text{c}}^{-}$ (Qin et al., 2006). Other found that it also induced L-glu release from astrocytes via activation of the α7 nicotinic acetylcholine receptor (Talantova et al., 2013). In addition, xCT, the specific subunit of system x${}_{\text{c}}^{-}$ is upregulated in the vicinity of senile plaques, probably in microglia, in Thy1-APP_751_ mice (TgAPP) expressing human APP bearing the Swedish (S: KM595/596NL) and London (L: V6421) mutations as well as after Aβ injection in the hippocampus (Qin et al., 2006). Semiquantitative immunoblot analysis revealed an upregulation of xCT protein expression in the frontal cortex in aged AβPP23 mice compared to wild-type controls (Schallier et al., 2011).

Postmortem studies indicate that KYN metabolism might also be modified in AD as increased concentrations of KYNA were found in the basal ganglia of AD patients (Baran et al., 1999). Using immunohistochemistry, Guillemin et al. found immunoreactivity for both IDO and QUIN upregulated in AD brains, especially in the vicinity of plaques (Guillemin et al., 2005). Of note, Aβ induces IDO expression in human primary macrophages and microglia (Guillemin et al., 2003). Indeed, systemic inhibition of KMO lead to increases in brain KYNA levels and ameliorated the phenotype of a mouse model of AD (Zwilling et al., 2011), indicating that an upregulation of KYNA might be an endogenous protective response. Also the IDO inhibitor, coptisine, reduced microglial, and astrocytic activation and cognitive impairment in AD mice (Yu et al., 2015).

**Figure 4:**
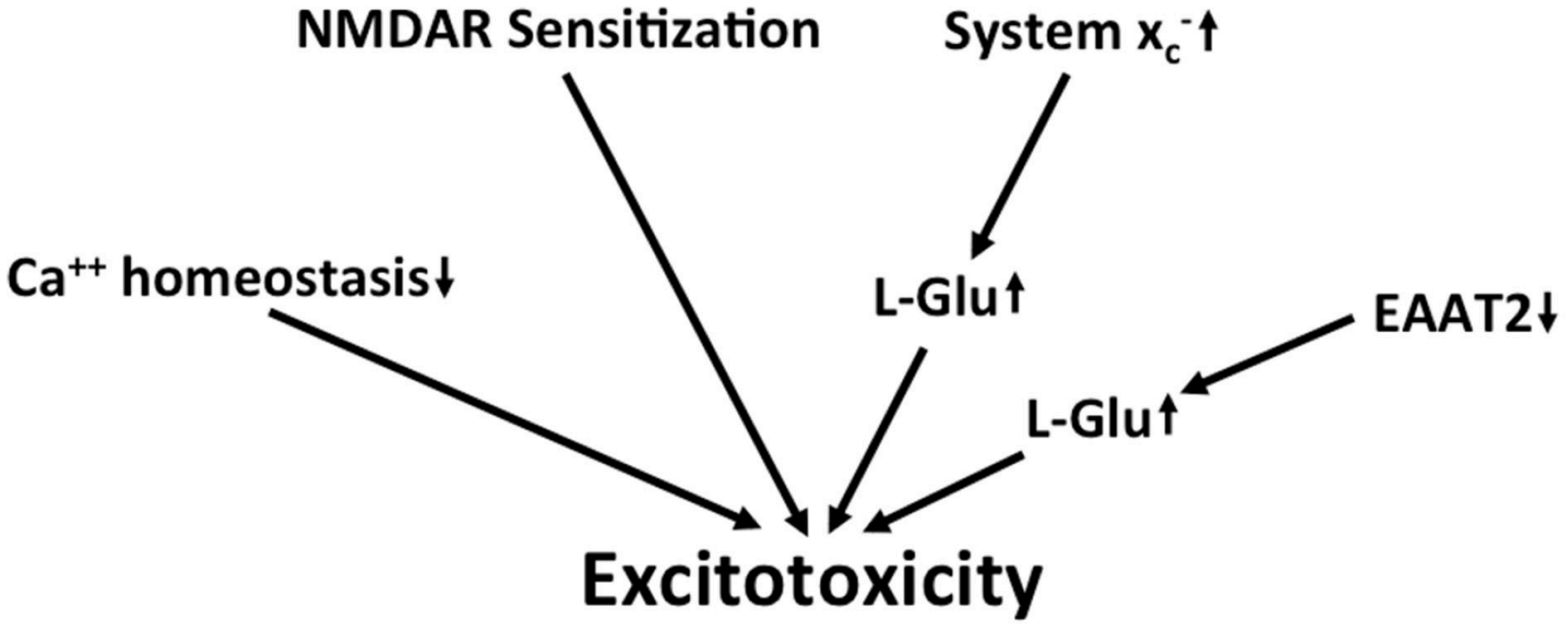
**Potential mechanisms that lead to excitotoxicity in AD**. Altered calcium homeostasis and increased sensitization of NMDA receptors in AD renders neurons more sensitive to excitotoxicity. This is further amplified by the upregulation of extracellular glutamate via downregulation of EAAT2 and upregulation of system x${}_{\text{c}}^{-}$.

Taken together, along with several other detrimental changes, there is evidence for chronic excitotoxicity in AD which may be driven by multiple factors including the sensitization of NMDA receptors, a decrease in L-glu and L-asp reuptake capacity and an increase in glutamate release via system x${}_{\text{c}}^{-}$ (Figure 4). Although the KYN pathway seems to be upregulated in AD, no specific conclusions can be drawn regarding glutamatergic neurotransmission from the reported upregulation of both neurotoxic QUIN and neuroprotective KYNA.

#### Huntington's disease

HD is a dominantly inherited, fatal neurodegenerative disease caused by a trinucleotide (CAG) repeat expansion in the coding region of the *huntingtin* (htt) gene that leads to the degeneration of GABAergic medium-sized spiny neurons (MSN) in the striatum, although other brain regions are also affected as the disease progresses. HD presents as a movement disorder with co-morbid psychiatric and cognitive symptomatology (Nance, 1997). Both mutant htt RNA and the encoded protein that contains a polyglutamine repeat expansion are thought to lead to complex alterations in cellular metabolism culminating in mitochondrial dysfunction and oxidative stress (Ayala-Peña, 2013; Johri et al., 2013; Tsoi and Chan, 2014).

Early findings that suggested that excitotoxicity might play an important role in HD were based on the observation that injection of the KYN metabolite and NMDA receptor agonist QUIN, as well as L-glu and kainate, into the striatum of rats generated neuronal degeneration (Coyle and Schwarcz, 1976; Beal et al., 1986). Beal et al. reported that QUIN, as compared to NMDA and kainate, induces a rather selective degeneration of the MSNs rather than general neuronal death (Beal et al., 1986), thus strongly resembling the pathology of HD. Later, NMDA receptors were found to be hyperactive and striatal neurons from different HD mouse models, including those transgenic for a yeast artificial chromosome (YAC) that leads to over-expression of full-length htt with extended polyglutamine repeats (Zeron et al., 2002; Shehadeh et al., 2006), R6/2 mice over-expressing htt exon 1 with extended polyglutamine repeats as well as in knock-in mice with increased CAG repeats inserted in the mouse htt gene (Levine et al., 1999), were shown to be sensitized to excitotoxicity *in vitro*. Of note, *in vivo,* a sensitization to excitotoxin injection into the striatum was only observed in the transgenic YAC model of HD (Zeron et al., 2002), whereas mice over-expressing mutant htt exon 1, R6/1 and R6/2 mice, (Hansson et al., 1999, 2001b) or N171-82Q mice over-expressing mutant exon 1 and parts of exon 2 (Jarabek et al., 2004) or the so-called “shortstop” mouse expressing human N-terminal htt encoded by exon 1 and 2 with a 128 CAG repeat under the htt promoter (Slow et al., 2005) actually developed resistance to striatal excitotoxin injection during aging. However, this acquired neuroprotection is not specific for NMDA receptor agonists but extends to other neurotoxic insults (Hansson et al., 2001a; Petersén et al., 2001) and may represent an adaptive response to cellular stress.

Rat MSN express high levels of NR2A- and NR2B-containing NMDA receptors when compared to interneurons in the striatum (Landwehrmeyer et al., 1995). Correspondingly, NR1 and NR2B mRNA expression in the neostriatum of HD patients was found to be prominently decreased which correlated with the loss of the neurons (Arzberger et al., 1997). Moreover, NMDA receptor-mediated currents in MSN were demonstrated to be largely sensitive to the NR2B-specific inhibitor ifenprodil (Zeron et al., 2002). In HEK293 cells, over-expression of mutant htt increased NMDA receptor-mediated currents and exacerbated NMDA-induced cell death only when NR2B- but not when NR2A-containing NMDA receptors were co-expressed (Chen et al., 1999; Zeron et al., 2001). One possible explanation for the increase in NR2B-containing NMDA receptor expression in HD models is that an extended polyglutamine repeat in htt decreases its binding to PSD95, a postsynaptic density protein involved in NMDA and kainate receptor clustering, leading to increased interaction of PSD95 with the NR2B subunit (Sun et al., 2001; Fan et al., 2009). More recently, data have been presented that suggest that not only the subunit composition but also the localization of NMDA receptors might play an important role in the deleterious NMDA receptor activity in HD. Milnerwood et al. (2010) showed that in acute striatal slice preparations from YAC transgenic mice with 128 CAG repeats, extrasynaptic NMDA receptors, especially those containing NR2B, are significantly increased compared to slices from wild-type mice and YAC mice expressing htt with 18 CAG repeats. As expected from *in vitro* studies (Hardingham et al., 2001), this change was associated with reduced CREB phosphorylation (Milnerwood et al., 2010). The increased proportion of NR2B-containing extrasynaptic NMDA receptors was demonstrated to be associated with an increased extrasynaptic localization of PSD95 (Fan et al., 2012). One pathway that might mediate the sensitization to excitotoxic stimuli downstream of the activation of extrasynaptic NMDA receptors was identified as activation of p38 MAPK (Fan et al., 2012). Taken together, multilayered evidence suggests that mutant htt leads to sensitization of MSN to glutamate excitotoxicity in part through a relative redistribution of NMDA receptors, especially those containing an NR2B subunit, from synaptic to extrasynaptic sites.

The activation of extrasynaptic NMDA receptors in acute striatal brain slices can be effectively induced in YAC mice with 128 CAG repeats by facilitating spillover of synaptic glutamate by inhibiting EAATs (Milnerwood et al., 2010). Consequently, it can be hypothesized that decreased EAAT expression might increase activation of extrasynaptic NMDA receptors. Interestingly, using *in situ*-hybridization, Arzberger et al. found a decrease in astrocytic EAAT2 mRNA expression in the neostriatum of HD patients (Arzberger et al., 1997). EAAT2 function was found to be decreased in striatal synaptosomes of YAC mice over-expressing human htt with 128 CAG repeats as compared to wild-type mice, however no changes in EAAT2 protein expression could be detected. The authors argued that a functionally relevant decrease in EAAT2 activity in the YAC model of HD was due to reduced palmitoylation of the transporter (Huang et al., 2010). In R6/2 mice, others found reduced EAAT2 mRNA and protein expression (Liévens et al., 2001; Behrens et al., 2002; Shin et al., 2005; Estrada-Sánchez et al., 2009) associated with decreased EAAT2 function in synaptosomes (Liévens et al., 2001) or acute cortico-striatal slices (Shin et al., 2005). However, extracellular striatal glutamate concentrations were found to be similar to those of wild-type control mice (Gianfriddo et al., 2004; Shin et al., 2005) and a reduced glutamate clearance capacity in the R6/2 mice could only be revealed by treatment with EAAT inhibitors or glutamate (Behrens et al., 2002; Estrada-Sánchez et al., 2009). A putative explanation for this finding could be a decrease in glutamate release via system x${}_{\text{c}}^{-}$ as recently a decrease in xCT, the specific subunit of system x${}_{\text{c}}^{-}$, was demonstrated in the striatum of R6/2 mice at the mRNA and protein levels (Frederick et al., 2014).

As mentioned above, injection of the KYN metabolite QUIN at supraphysiological concentrations was used as an early animal model of HD (Beal et al., 1986). This led to further investigations of KYN metabolism in HD. Interestingly, the QUIN precursor 3HK exacerbates neurodegeneration in the QUIN HD model (Guidetti and Schwarcz, 1999), while KYNA is protective (Foster et al., 1984; Schwarcz and Pellicciari, 2002). Guidetti et al. found that in early-stage HD, as compared to control and end-stage HD, neostriatal 3HK and QUIN concentrations were significantly upregulated (Guidetti et al., 2004). Another study found KYNA levels decreased in autopsied HD striata as well as in the CSF of HD patients as compared to controls (Beal et al., 1992). The initial enzyme of the KYN pathway, IDO, is induced in the striatum of YAC mice with 128 CAG repeats (Mazarei et al., 2013a). Mice deficient in IDO are less sensitive to intrastriatal QUIN injection (Mazarei et al., 2013b). Analysis of KYN metabolites in different brain regions from three different mouse models of HD, R6/2 mice, YAC128 mice, and HdhQ92 and HdhQ111 knock-in mice, suggested age-dependent activation of the KYN pathway. However, the detailed pattern of metabolite changes differed somewhat among the models with increased 3HK in cortex, striatum, and cerebellum in R6/2 mice whereas mice expressing full-length mutant htt showed an additional cortical and striatal upregulation of QUIN (Guidetti et al., 2006). Moreover, treatment of R6/2 mice with a non-blood brain barrier permeable KMO inhibitor, JM6, which indirectly increased cerebral extracellular KYNA concentrations by ~50%, was associated with a decrease in extracellular cerebral L-glu, decreased neurodegeneration and prolonged survival (Zwilling et al., 2011).

**Figure 5:**
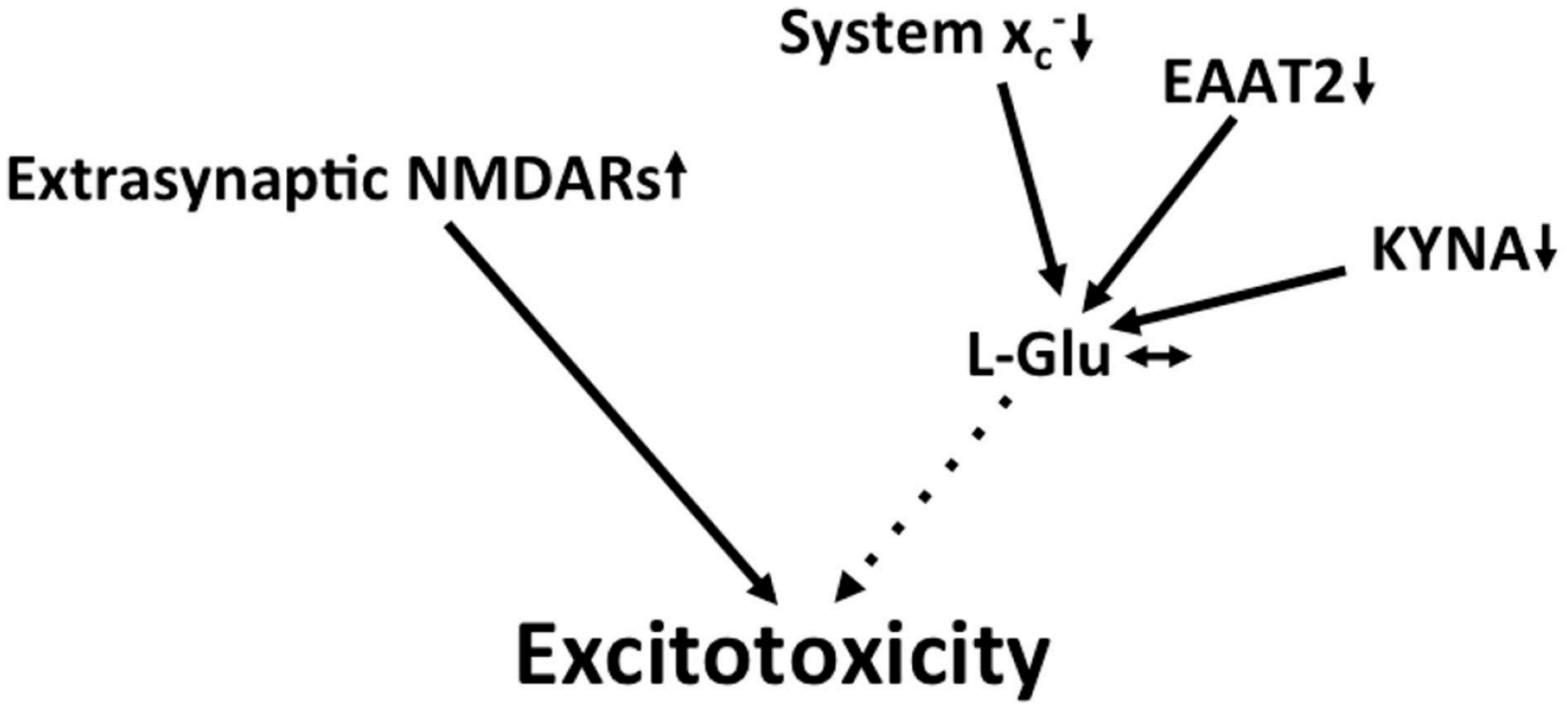
**Potential mechanisms that lead to excitotoxicity in HD**. Increased redistribution of NMDA receptors to the extrasynaptic compartment is thought to be the prevailing mechanism that fosters excitotoxicity in HD. Although EAAT2 and the glutamate-lowering kynurenine metabolite kynurenic acid (KYNA) are downregulated, these changes might be compensated for by a decrease in system x${}_{\text{c}}^{-}$ expression.

Taken together, the published literature supports the view that in HD there is a redistribution of NMDA receptors, especially those containing NR2B, which might activate signaling pathways that foster neurodegeneration (Figure 5). There is no consistent evidence that extracellular cerebral L-glu levels are grossly increased in HD. This might be explained by the fact that although EAAT2 and KYNA might be downregulated, there is also a downregulation of system x${}_{\text{c}}^{-}$ activity. As only very high levels of QUIN activated NMDA receptors, this KYN metabolite is unlikely to directly contribute to the excitotoxic load.

## Proof of concept experiments that support the hypothesis of chronic glutamate toxicity in neurodegeneration

As EAATs have been found to be down-regulated in many diseases of the nervous system (Sheldon and Robinson, 2007) and hypothetically increased L-glu and L-asp clearance should dampen the excitotoxic component of these diseases, many researchers have set out to identify compounds that induce EAAT2, which is the principal EAAT in the brain and most frequently found to be downregulated (Sheldon and Robinson, 2007; Kim et al., 2011). This has led to the identification of many compounds that *in vitro* (Colton et al., 2010; Xing et al., 2011) or both *in vitro* and *in vivo* (Rothstein et al., 2005; Ganel et al., 2006; Kong et al., 2014) induce astrocytic EAAT2 expression. Some of these have proven to be protective in animal models of neurodegenerative diseases (Rothstein et al., 2005; Ganel et al., 2006; Kong et al., 2014). Cef is perhaps the best studied of these compounds and has been tested in models of AD (Zumkehr et al., 2015), HD (Miller et al., 2008), and ALS (Rothstein et al., 2005) with positive results. However, it has to be kept in mind that none of these compounds has been extensively screened for its ability to interact with other cellular pathways that might also be neuroprotective. Of note, Cef has been shown not only to induce EAAT2 expression but also to activate the transcription factor Nrf2 (Lewerenz et al., 2009), which induces the transcription of a plethora of genes involved in cytoprotection and antioxidant defense (Kensler et al., 2007). Since oxidative stress is assumed to play a role in many, if not all, neurodegenerative diseases (Bogdanov et al., 2001; Radi et al., 2014), this pathway may account for at least some of the neuroprotection induced by Cef. Indeed, xCT, which is one of the downstream targets of Nrf2, has been found to be upregulated by Cef *in vitro* and *in vivo* (Lewerenz et al., 2009; Knackstedt et al., 2010). Another *in vitro* EAAT2-inducing compound, MS-153, effectively protected against secondary neurodegeneration after traumatic brain injury, but through mechanisms other that EAAT2 upregulation (Fontana et al., 2015). Thus, proof of concept experiments that unequivocally demonstrate the pathophysiological role of a chronically increased excitotoxic input via iGluRs in neurodegenerative diseases require more specific manipulations of the neurotransmitter physiology.

As described in the Section on “The Concepts of Acute and Chronic Glutamate Toxicity,” Glud1 Tg mice represent a model of chronic excitotoxicity mediated by increased synaptic L-glu release with limited neuronal loss (Bao et al., 2009). However, this animal model of increased glutamatergic neurotransmission has not yet been used to test whether Glud1 over-expression exacerbates the phenotype of mouse models of neurodegenerative diseases. Another genetically engineered model is the EAAT2-deficient mouse. As described in the Section on “The Concepts of Acute and Chronic Glutamate Toxicity,” homozygous EAAT2 knock-out mice suffer from premature death due to epilepsy and show hippocampal and focal cortical atrophy (Tanaka et al., 1997; Kiryk et al., 2008; Petr et al., 2015). Heterozygous EAAT2 knock-out mice however develop normally and show only mild behavioral abnormalities (Kiryk et al., 2008). Consequently, this mouse model of mild glutamatergic hyperfunction has been used in a series of proof of principle studies that investigated the functional role of glutamate in animal models of neurodegenerative diseases. ALS mice that carry both the G93A mSOD1 mutation and a reduced amount of EAAT2 (SOD1(G93A)/EAAT2^±^) exhibited an increase in the rate of motor decline accompanied by earlier motor neuron loss when compared with single mutant G93A mSOD1 Tg mice (Pardo et al., 2006). A modest reduction in survival was also noted in these double mutant mice. When crossed with transgenic mice expressing mutations of the human amyloid-β protein precursor and presenilin-1 (AβPPswe/PS1ΔE9), partial loss of EAAT2 unmasked spatial memory deficits in 6-month-old mice expressing AβPPswe/PS1ΔE9. These mice also exhibited an increase in the ratio of detergent-insoluble Aβ42/Aβ40 suggesting that deficits in glutamate transporter function contribute to early pathogenic processes associated with AD (Mookherjee et al., 2011). In contrast, the phenotype of the R6/2 HD mouse model was not altered in mice that contained only one EAAT2 allele (Petr et al., 2013).

As a complement to these studies, transgenic mice that over-express EAAT2 specifically in astrocytes via the GFAP promoter have also been developed (Guo et al., 2003). EAAT2/G93A mSOD1 double Tg mice showed moderate amelioration of the ALS-like phenotype with a statistically significant (14 days) delay in grip strength decline and loss of motor neurons as well as a reduction in other events including caspase-3 activation and SOD1 aggregation but not in the onset of paralysis, body weight decline or an extended life span when compared with monotransgenic G93A mSOD1 littermates (Guo et al., 2003). The same EAAT2 transgenic mouse model was used to test the effect of increased astrocytic L-glu (and L-asp) uptake by cross-breeding with an animal model of AD, AβPPswe/Indmice. Increased EAAT2 protein levels significantly improved cognitive function, restored synaptic integrity, and reduced amyloid plaques in these AD mice (Takahashi et al., 2015).

In mice in which genetically engineered deletion of xCT leads to deficiency in the glutamate/cystine antiporter system x${}_{\text{c}}^{-}$, the prominent reduction of extrasynaptic L-glu is associated with a robust resistance of dopaminergic neurons against 6-hydroxydopamine-induced neurodegeneration (Massie et al., 2011), possibly as a result of decreased excitotoxicity. However, microglial activation has also been shown to be modulated by system x${}_{\text{c}}^{-}$ deficiency resulting in a more neuroprotective phenotype (Mesci et al., 2015) which provides an alternative explanation for the protective effect of xCT deletion in this context.

Thus, genetic models support the role of chronic excitotoxicity in neurodegenerative diseases, especially ALS and AD. Of note, all of these models represent life-long changes in glutamatergic neurotransmission. From the therapeutic perspective, these models cannot predict whether drugs that specifically ameliorate the glutamatergic tone during the neurodegenerative process are protective. To this end, either intensive testing of EAAT2-inducing drugs for their interaction with other signaling pathways or the development of inducible mouse models with dampened excitotoxic load are warranted.

## Summary

In summary, “glutamatergic” excitatory input on neurons is a sum of the interaction of several different activators including the direct activators, L-glu, and L-asp, and the co-activators, glycine, and D-serine, on iGluRs and mGluRs. Other pathways including tryptophan metabolism and, especially, the tryptophan metabolite KYNA, modulate glutamatergic neurotransmission. Glutamatergic input on neurons is either via synaptically released L-glu (and L-asp) acting on synaptic iGluRs or by non-synaptically released L-glu acting at extrasynaptic L-glu receptors. The glutamate/cystine antiporter, system x${}_{\text{c}}^{-}$, might be an important determinant of the extrasynaptic cerebral L-glu concentration. Chronically increased input via iGluRs, even if it is only moderate, has the propensity to induce neuronal degeneration, so-called chronic excitotoxicity. In many neurodegenerative diseases, including HD, AD, and ALS, multilayered evidence suggests that glutamatergic dysregulation is an important contributor to disease pathology although the molecular basis for this varies widely and might be distinct for each disease and most likely does not represent the only pathway that leads to neurodegeneration.

However, as specific pharmacological tools or inducible genetically engineered mouse models that allow manipulation of glutamatergic input are lacking, it is not known to what extent L-glu dysregulation contributes to disease progression in specific mouse models of different neurodegenerative diseases. Thus, while the idea that chronic excitotoxicity contributes to multiple neurodegenerative diseases is supported by many layers of scientific evidence, it is not clear that therapeutic interventions that re-establish glutamatergic homeostasis during ongoing neurodegeneration will be effective tools for stopping the disease process. Besides direct modulators of iGluR activity, strong candidates for future approaches to treating chronic excitotoxicity include specific inducers of EAAT2 to stimulate L-glu and L-asp uptake, inhibitors of system x${}_{\text{c}}^{-}$ to reduce L-glu release as well as compounds that aim to decrease extracellular L-glu by modulating KYN metabolism, e.g., KMO inhibitors. In addition, it has to be kept in mind that combinations of these interventions might be required to obtain clinically significant benefits without evoking adverse side effects.

## Author contributions

JL provided the concepts for the review and wrote much of it. PM wrote a portion of the review and edited the entire review.

## Funding

PM was partially supported by NIH.

### Conflict of interest statement

The authors declare that the research was conducted in the absence of any commercial or financial relationships that could be construed as a potential conflict of interest.

## Abbreviations

Aβ: amyloid beta
AD: Alzheimer's disease
ADME: absorption, distribution, metabolism and excretion
ALS: amyotrophic lateral sclerosis
ALS/PDC: Guamian amyotrophic lateral sclerosis/Parkinson-dementia complex
AMPA: α-amino-3-hydroxy-5-methyl-4-isoxazolepropionic acid
ANA: anthranilic acid
APP: amyloid precursor protein
Asc-1: alanine-serine-cysteine amino acid transporter 1
L-asp: aspartate
BMAA: β-*N*-methylamino-L-alanine
BOAA: β-*N*-oxalylamino-L-alanine
Cef: ceftriaxone
CREB: cyclicAMP response element-binding protein
CSF: cerebral spinal fluid
CNS: central nervous system
DAO: D-amino acid oxidase
DIV: days *in vitro*
EAAT: excitatory amino acid transporter
GFAP: glial fibrillary acidic protein
iGluR: ionotropic glutamate receptor
L-glu: glutamate
3HANA: 3-hydroxyanthranilic acid
3-HAO: 3-hydroxyanthranilate oxidase
L-HCA: homocysteate
HD: Huntington's disease
3HK: 3-hydroxykynurenine
Htt: huntingtin protein
IDO: indoleamine 2, 3-dioxygenase
KAT: kynurenine aminotransferase
KMO: kynurenine monoxygenase
KYN: kynurenine
KYNA: kynurenic acid
MAPK: mitogen activate protein kinase
mGluR: metabotropic glutamate receptors
MSN: medium spiny neurons
NMDA: N-methyl-D-aspartic acid
QUIN: quinolinic acid
SOD1: superoxide dismutase 1
SSZ: sulfasalazine
TBI: traumatic brain injury, Tg, transgenic, TSC, tuberous sclerosis complex
vGLUT: vesicular glutamate transporters
YAC: yeast artificial chromosome.
